# Caloric Vestibular Stimulation Induced Enhancement of Behavior and Neurotrophic Factors in Chronic Mild Stress Induced Rats

**DOI:** 10.3389/fphar.2022.834292

**Published:** 2022-04-27

**Authors:** Sherly Deborah George, Rajagopalan Archana, Subramani Parasuraman

**Affiliations:** ^1^ Department of Physiology, Faculty of Medicine, Manipal University College Malaysia, Melaka, Malaysia; ^2^ Department of Physiology, Saveetha Medical College, Saveetha Institute of Technical and Medical Sciences (SIMATS), Chennai, India; ^3^ Unit of Physiology, Faculty of Medicine, AIMST University, Kedah, Malaysia; ^4^ Department of Pharmacology, Faculty of Pharmacy, AIMST University, Kedah, Malaysia

**Keywords:** caloric vestibular stimulation, chronic mild stress, brain derived neurotrophic factors, glial cell line-derived neurotrophic factors, behavior

## Abstract

**Background:** Caloric Vestibular Stimulation (CVS) is a non-invasive technique for stimulating the vestibular system. The vestibular system maintains equilibrium and acts as a moderator of mood, emotional control, and stress levels. Stress is a disruption of psychological, behavioral, and physiological homeostasis that affects people of all ages in today’s world. Thus, modest therapeutic procedures like vestibular stimulation can be practiced to effectively reduce stress. Hence, the purpose of the study was to determine the effect of vestibular stimulation on improving behavioral alterations and neurotrophic factors in rats exposed to **Chronic Mild Stress (CMS)**.

**Methodology:** The study employed 24 healthy male Sprague Dawley rats divided into four groups (*n* = 6). CMS was induced for 28 days with a variety of stimuli. Bilateral CVS with hot water (temperature ≈40°C) was started on Day 14 of CMS and continued for 15 days. On days 1, 15, and 28, locomotor activity (LA), wire grip strength (WGS), fall off time (FT), and immobilization time (IT) were measured, and the data were analyzed statistically. Additionally, neurotrophic factors such as Brain Derived Neurotrophic Factor (BDNF) and Glial cell line-Derived Neurotrophic Factor (GDNF) were observed in rats’ hippocampus.

**Results:** On days 15 and 28, the CMS-induced group showed a significant reduction in LA, WGS, FT and IT in comparison to the control group. On day 28, the CVS-induced group demonstrated a significant increase in WGS, FT and IT when compared to the CMS group. Immunohistochemical analysis revealed that animals subjected to CMS had decreased BDNF and GDNF expression compared to the control group, indicating neuronal dysfunction in the hippocampus in response to stress. However, therapy with CVS increased BDNF and GDNF expression, thereby regenerating damaged hippocampus nerve terminals.

**Conclusion:** The findings of the current study revealed that CVS is a safe and simple neuroprotective treatment against stress and a promising non-invasive technique for overcoming the motor symptoms associated with it. The findings may pave the way for future research and therapeutic applications of CVS for stress management.

## Introduction

The last century, stress has become an inevitable aspect of our lives, and its incidence rate has soared, particularly among the younger generations. One-third of the global population has reported feeling stressed, and the number is increasing constantly^1^. Stress is depicted as a change in psychological, behavioral, and physiological equilibrium (Bekris et al., 2005) which causes the “wear and tear” of the body when it responds to pressure or a potentially dangerous circumstance (Almojali et al., 2017). The stimulation of the hypothalamic-pituitary-adrenal (HPA) axis is one of the essential systems reacting to stress to guarantee an optimal response to stress. Chronic stress, which is linked to hippocampal alterations, may be linked to the inception of psychotic ailments (Kajantie and Phillips, 2006). The Chronic Mild Stress (CMS) model of depression (Willner et al., 1992; Willner, 1997) is generally regarded as a paradigmatic example and has been shown to generate an anhedonic-like state in rats, which mirrors some stress symptoms in humans (Bekris et al., 2005) and may thus be applied to gain a better understanding of human psychopathology (Willner, 2017).

The vestibular system, which regulates posture and equilibrium, is inextricably linked to the whole physiology of the body (Goldberg et al., 2012). Vestibular information is transmitted to the hippocampus through brain regions that receive vestibulo-thalamocortical projections, such as the parietal cortex. According to electrophysiological observations, the brainstem vestibular nucleus complex and the hippocampus were polysynaptically coupled, and hippocampal cells responds to vestibular stimulation. Damage to the vestibular system has a long-term influence on the hippocampus’s electrophysiological and neurochemical function (Smith et al., 2005). Vitte *et al* used caloric vestibular stimulation (CVS) to explore the changes in blood oxygenation in the hippocampus with magnetic resonance imaging, and he established direct evidence for vestibular-hippocampal communication in humans. They concluded that CVS engaged the hippocampus mostly ipsilaterally, as well as Brodmann’s area 39–42, the posterior insular cortex, Brodmann’s area 7 in the superior parietal lobe, and the retrosplenial cortex and subiculum (Vitte et al., 1996).

The vestibular system also helps with a wide range of tasks, from reflexes to cognition and coordination. As a result, vestibular sense is sometimes known as “The Sixth Sense” (Baizer et al., 2013). The controlled stimulation of the vestibular system has consistently been applied for neurological diagnosis (Miller and Ngo, 2007) and has proven to be beneficial in the treatment of dementia (Kranthi et al., 2021), regulation of brain ageing neurotransmitters (Nishiike et al., 1997), and the alleviation of depression and anxiety (Kranthi et al., 2021). However, studies relating vestibular stimulation to behavior and neurotrophic factors in stress are sparse. Therefore, the current study was intended to assess the effect of vestibular stimulation on behavior and neurotrophic factors in CMS-induced rats.

## Materials and Methods

### Reagents

The materials used in this study were primary antibody: Anti-BDNF (ab108319) and anti- GDNF (ab18956) which were purchased from Abcam, United States. Secondary antibody: Rabbit specific HRP_DAB (horseradish peroxidase_ 3,3′ Diaminobenzidine) IHC Detection Kit - Micro-polymer was bought from Abcam, United States. Antigen retrieval buffer was obtained from Abcam, United States. Bovine Serum Albumin (BSA), Tris- Ethylenediaminetetraacetic acid (EDTA) buffer and xylene were purchased from Sigma, United States. Tris-Buffered Saline, 0.05% Tween 20 (TBST), Phosphate Buffered Saline (PBS), Phosphate Buffered Saline plus Tween 20 (PBS-T), Diamidine-2′-phenylindole dihydrochloride (DAPI), 10% paraformaldehyde, paraffin, hydrogen peroxide was purchased from Eman Biodiscoveries Sdn Bhd, Penang, Malaysia.

### Experimental Animals

The study employed healthy, adult, male Sprague Dawley (SD) rats weighing 180 ± 20 g. The rats were housed and maintained in large, spacious polyacrylic cages with a 12-h light/12-h dark cycle maintained at ambient room temperature. Water and standard rat pellet food were provided *ad libitum* to the animals. The study was approved by the University Human and Animal Ethics Committee (AUAEC/FOM/2020/03) and was conducted in accordance with the requirements of the Animal Research Review Panel guidelines. All behavioral experiments were conducted between 9.00 am and 12.00 p.m.

### Chronic Mild Stress (CMS) Administration

The CMS model employed in this study is a modified version of the CMS approach used in the earlier studies (Willner, 2017). Various stressors with varying durations were applied continuously for 28 days. Throughout these 28 days, the control group animals were not exposed to CMS. Each day, the following stressors were introduced one by one, and the cycle was repeated.

*Restraint Stress:* For an hour, rats were placed in a restraining device constructed of flexible nylon, limiting movement but permitting free respiration and air circulation.

*Overcrowding:* Overnight placement of two home-cages of rats from the same experimental group in a single cage.

*Wet bedding:* Rats were wetted by placing them in a cage with wet husk (5 cm high) for 5 h.

*Forced swimming:* Each rat was placed in a 1-L cylindrical plastic Baxter container full of 850 ml tap water (24–26°C) and forced to swim for 5 min.

*Tail pinch in restrainer:* For 20 min, the rat was placed in the previously described restraining device and a clothespin was fastened 2 cm from the base of the tail.

*Cold water swim test*: The rat was placed in a cylindrical tank (60 cm height × 30 cm diameter) filled with water to a depth of 30 cm at 8°C for 5 min.

*Inversion of the day and night cycle:* Cages were housed in a separate dwelling, and the inversion of the day and night cycle was retained.

### Caloric Vestibular Stimulation (CVS)

The animal was placed in a rat restrainer and the middle ear cavity of each ear was irrigated with a syringe filled with 2 ml of warm water at 42 ± 2°C. Constant vestibular stimulation was achieved by maintaining the flow rate at 0.1 ml/s. To generate convection currents in the semicircular canals, the rats were positioned such that the horizontal canal was tilted approximately 30° degrees with regard to the horizontal plane (Nishiike et al., 1997; Nishiike et al., 2000).

### Experimental Design

The experimental rats were randomly divided into four different groups (each group consisting of six animals).

Group 1 – Control.

Group 2 – CMS.

Group 3 – CVS.

Group 4 – CMS + CVS.

The group I animals were normal animals and free from CVS or CMS. The animals in group II were induced with CMS once daily for 28 days. The animals in group III were given CVS treatment, once daily for 15 days and the animals in group IV were induced with CMS once daily for 28 days and were given CVS treatment from day 15 onwards i.e., the rats were stressed for 28 days (verified by behavioral changes), and on the day 15, CVS was delivered for 15 days concurrently with stress induction. At the end of the study, the animals were sacrificed, and the brain sample was collected for immunohistochemical analysis.

### Behavioral Assessment

On pre-study day, day 14 and day 28, locomotor activity, muscular coordination, grip strength, and immobilization time were evaluated.

#### Locomotor activity

The actophotometer (rat activity cage) was used to record the locomotor activity of the rats. It is comprised of an acrylic cage and eight infrared light beams along both the *x* and *y* axis. At room temperature, each rat’s activity was recorded for 10 min (Parasuraman et al., 2020).

#### Rotarod test

The motor coordination of the rat was evaluated using rotarod instrument. The instrument is composed of a rotating horizontal metal rod. All rats were trained for 5 days before the experiment could start. On the first day of training, the rotation speed was set to 17 rpm and gradually increased to 20 rpm on the last day. Three trials were conducted on the rat, with a 5-min break in between. The time interval between the rat’s retention on the rotarod and its fall was recorded. Each rat was subjected to the same technique. As a reading, the mean of three trials was considered (Kishore Kumar et al., 2017; Parasuraman et al., 2020).

#### Hanging wire Grip strength test

The metallic wire string was linked horizontally to two vertically arranged rods. The rat was placed in the center of the string, with its forelimb clinging to the string while its body and tail were suspended in the air over 30 cm from the ground. The distance between the rope and the ground was kept as low as possible to prevent injuring the rodent during its fall. The time required for the rat to fall was recorded, i.e., “fall off time” (Parasuraman et al., 2020).

#### Forced swimming test

Forced swimming test was used to measure the immobilization time. The tank was filled with water in a temperature range of 24–30°C to ensure that the rodents’ tails and feet did not contact the bottom. After that, the rat was placed in the tank. The time interval between the rodent ceasing to swim and beginning to sink was recorded. As soon as the rodent began to sink, it was promptly removed. All rodents were subjected to the same treatment. The water tank was subsequently cleansed whenever excrement and urine accumulated, as this could result in bacterial contamination (Palanimuthu et al., 2016).

### Brain Tissue Pre-processing

After 28 days of intervention, the rats were euthanized by cervical dislocation, and the hippocampus was dissected from fresh rat brain and transferred to tissue cassettes immersed in 10% neutral buffered formalin (Sigma) for overnight fixing. Following fixation, the cassettes were transported to a Thermo tissue processor for 16 h according to manufacturer’s protocol, which included fixation, dehydration, clearing, and wax infiltration. Tissues were imbedded in wax (Thermo) and chilled to room temperature before being trimmed into 4 µm sections using a Leica microtome. The sections were fished out using Poly-Lysin coated slides (Thermo) for Immunohistochemistry **(**IHC) staining. All prepared slides were dehydrated at room temperature (Badroon et al., 2020; Al-Suede et al., 2021).

### Determination of the Expression of Brain Derived Neurotrophic Factor (BDNF) and Glial Cell Line-Derived Neurotrophic Factor (GDNF) in Hippocampus by Using IHC Technique

Brain tissues were harvested and preserved in a 10% paraformaldehyde solution. The paraffin-embedded tissue block was sliced into 4 μm sections. The tissue was deparaffinized and rehydrated prior to IHC staining using a graded series of ethanol. The sections were incubated in a microwave with antigen retrieval buffer and then rinsed with TBST (1×), followed by a 10-min incubation with hydrogen peroxide block. Following that, the section was blocked with 5% BSA for 10 min and incubated overnight at 2°C with primary antibodies (anti-BDNF or anti-GDNF), followed by washing with PBS. For 10 min at room temperature, sections were treated with secondary antibody (Rabbit specific HRP DAB). The negative control sections received the identical treatment as the positive control sections, but without the addition of primary antibodies. Sections were washed three times in PBS-T followed by mounting with DAPI (Fluorescent mounting medium). Finally, sections were examined under a microscope with ×100 objective. The percentage of BDNF and GDNF protein expression was measured using imageJ software (Badroon et al., 2020; Al-Suede et al., 2021).

### Statistical Analysis

The mean and standard error of the mean (SEM) were used to express descriptive data. Statistical significance was fixed at *p* value less than 0.05 for all behavioral tests. The data for behavioral studies were analyzed using one-way ANOVA followed by Tukey’s *post hoc* test.

## Results

### Effect of CVS on the Behavior of Rats

**FIGURE 1:**
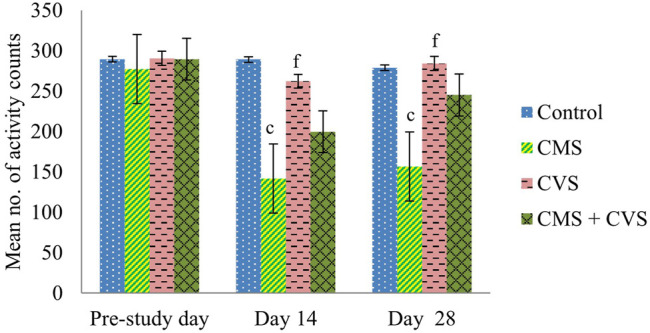
Effect of CVS on locomotor activity. Values are expressed as mean ± SEM (*n* = 6). ^c^ *p* < 0.001 compared with that of control group; ^f^ *p* < 0.001 compared with that of CMS group. (One-way ANOVA followed by Tukey’s *post hoc* test).

**FIGURE 2:**
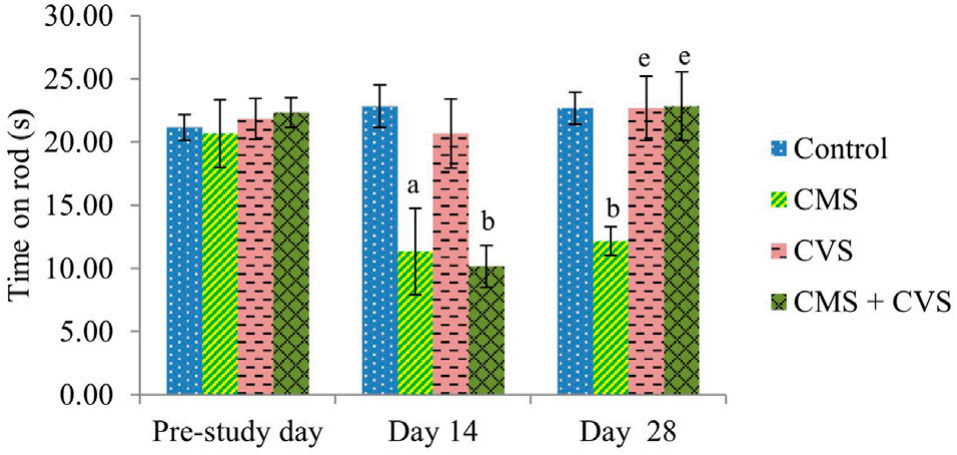
Effect of CVS on fall off time. Values are expressed as mean ± SEM (*n* = 6). ^a^ *p* < 0.05, ^b^ *p* < 0.01 compared with that of control group; ^e^ *p* < 0.01 compared with that of CMS group. (One-way ANOVA followed by Tukey’s *post hoc* test).

**FIGURE 3:**
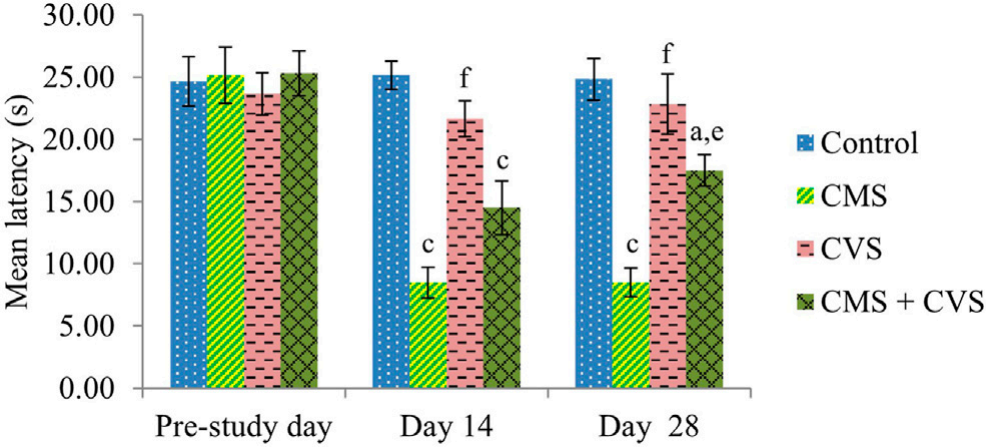
Effect of CVS on wire grip. Values are expressed as mean ± SEM (*n* = 6). ^a^ *p* < 0.05, ^c^ *p* < 0.001 compared with that of control group; ^e^ *p* < 0.01, ^f^ *p* < 0.001 compared with that of CMS group. (One-way ANOVA followed by Tukey’s *post hoc* test).

**FIGURE 4:**
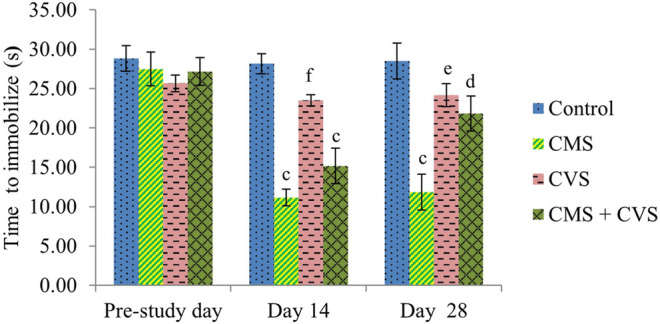
Effect of CVS on immobilization time. Values are expressed as mean ± SEM (n = 6). ^c^ *p* < 0.001 compared with that of control group; ^d^ *p* < 0.05, ^e^ *p* < 0.01, ^f^ *p* < 0.001 compared with that of CMS group. (One-way ANOVA followed by Tukey’s *post hoc* test).

At the end of the study, when compared to control rats, CMS-induced rats had significantly lowered locomotor activity (Figure 1), motor coordination (Figure 2), muscle grip strength (Figure 3), and immobilization time (Figure 4) whereas the CMS-induced rats which were administered CVS showed a significant increase in locomotor activity (Figure 1), motor coordination (Figure 2), muscle grip strength (Figure 3), and immobilization time (Figure 4) when compared to the CMS group. The rats which were administered CVS alone showed no significant differences in locomotor activity (Figure 1), muscle grip strength (Figure 3), or immobilization time (Figure 4) when compared to control rats.

### Effect of CVS on BDNF Levels in CMS-Induced Rats

**FIGURE 5:**
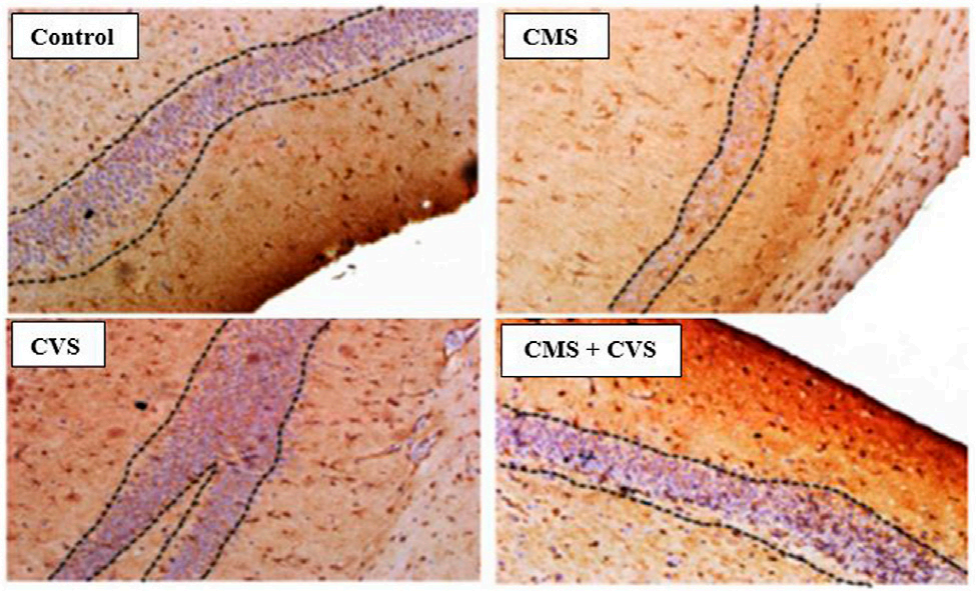
Immunohistochemical staining for BDNF in rat hippocampus section. Shown are representative photomicrographs of different groups of rats. BDNF staining is prominently visible in the sections as dotted lines.

**FIGURE 6:**
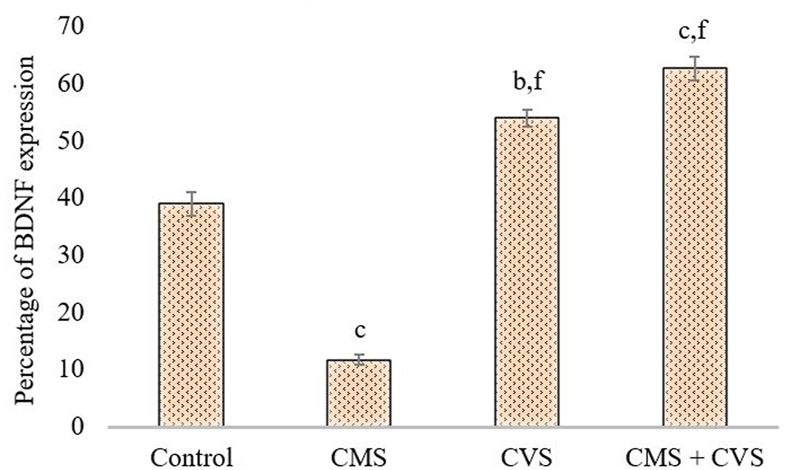
Quantitative immunohistochemical analysis of BDNF. Values are expressed as mean ± SEM (*n* = 6). ^b^ *p* < 0.01, ^c^ *p* < 0.001 compared with that of control group; ^f^ *p* < 0.001 compared with that of stress group. (One-way ANOVA followed by Tukey’s *post hoc* test).

The immunohistochemical staining for BDNF protein is clearly apparent in the sections of the control group, demonstrating that BDNF is expressed normally and constitutively in the hippocampus of the control animals (Figure 5). CMS-exposed rats, on the other hand, had lower levels of BDNF expression than the control group. Among the treated groups, CMS+CVS animals had the highest level of BDNF protein expression, followed by the group which was given CVS which is evident with the quantitative analysis (Figure 6).

### Effect of CVS on GDNF Levels in CMS-Induced Rats

**FIGURE 7:**
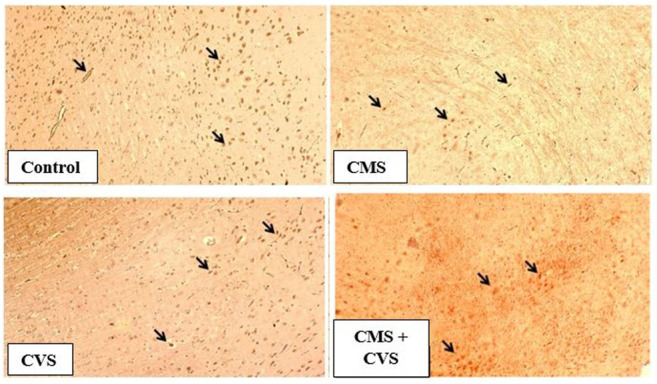
Immunohistochemical staining for GDNF in rat hippocampus section. Shown are representative photomicrographs of different groups of rats. GDNF staining is prominently visible in the sections marked with arrows.

**FIGURE 8:**
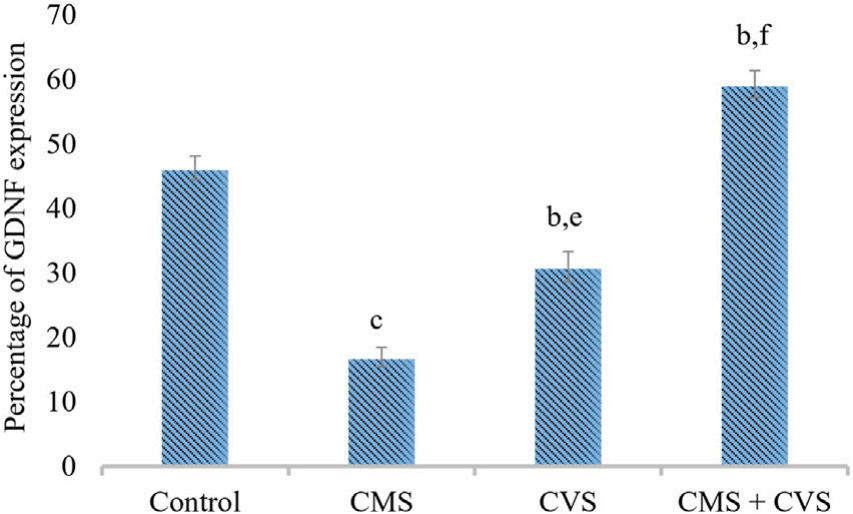
Quantitative immunohistochemical analysis of GDNF. Values are expressed as mean ± SEM (*n* = 6). ^b^ *p* < 0.01, ^c^ *p* < 0.001 compared with that of control group; ^e^ *p* < 0.01, ^f^ *p* < 0.001 compared with that of stress group. (One-way ANOVA followed by Tukey’s *post hoc* test).

The results showed that rats exposed to CMS had lower levels of GDNF protein expression in the hippocampus when compared to the control and treatment groups (Figure 7). When compared to the untreated group, the treated groups that received CVS and CMS+CVS showed a strongly significant increase in GDNF protein expression. GDNF expression was substantially higher in the CMS + CVS group than in the CMS group. Among the treated groups, CMS+CVS group had the highest level of GDNF expression, followed by the group administered with CVS only indicating that CVS enhances GDNF expression in the brain tissue as proven by quantitative analysis (Figure 8).

## Discussion

The current study was established to assess the protective role of CVS in CMS-induced rats in terms of behavioral changes and modifications of neurotrophic factors (BDNF and GDNF). In this study, CVS prevented the CMS-induced changes in locomotor behaviour, muscle grip strength and immobilization time. The results indicate that CVS can be helpful to modulate CMS-induced behavioral changes and improve the psychomotor function.

Stress affects the hippocampus, amygdala, and prefrontal cortex of the brain (Bremner, 2006), by stimulating the HPA axis, which is mediated through the hippocampus (Zhu et al., 2014). The principal stress-response mechanism is the HPA axis, which regulates various neurological functions at both the central and peripheral levels of the brain. Any type of stress has an impact on brain functions and can lead to a variety of neurodegenerative disorders (Esch et al., 2002; McEwen, 2004). The CMS model of stress is a prototypical example in which rats are subjected progressively to a series of severe stressors. In humans it can be associated to suffering from depression due to the normal unpredictable pressures of human existence (Willner, 2017). Hence, CMS model was employed to analyze the antidepressant-like effects of CVS in this study. As a result of chronic exposure to unpredictable micro-stressors, a cascade of behavioral changes ensue, all of which are linked to the clinical core symptom of stress. The symptoms of CMS reflect an enhanced physiological stress response. Previous experiments revealed that the glucocorticoid receptor antagonist mifepristone, the corticosterone synthesis inhibitor metyrapone, or adrenalectomy restrict the development of a depressive phenotype after CMS exposure. This demonstrates the vital relevance of HPA system in the impacts of CMS. The critical factor is that negative feedback systems operating through forebrain structures keep HPA activity in check, with the primary feedback occurring at the level of the hippocampus (Welberg and Seckl, 2001), and hence the levels of neurotrophic factors were assessed in the hippocampus which is an area of continued neurogenesis throughout the life of the brain. The hippocampus is a primary stress track owing to its role in regulating HPA axis function as well as its susceptibility to stress (Zaidi and Banu, 2004; Ansari et al., 2012). CMS exposure causes a preliminary activation of microglia, a marker of neuropathology that is triggered by increasing glucocorticoid exposure. The long-term exposure of the hippocampus to glucocorticoids disrupts cell metabolism by slowing glucose absorption and renders neurons more susceptible to metabolic stimuli (Sapolsky, 1987). Stress decreases the inhibitory input to the HPA axis, leading to overactivation of the HPA axis, which boosts the corticosterone level. The vestibular system has extensive connections with many cognitive parts of the brain, including the hippocampus, basal ganglia, parieto-frontal cortices, and cerebellum, which are referred collectively as vestibular cortices (Brandt, 2003). CVS can directly suppress the Sympatho-Adrenal-Medullary (SAM) and HPA axis by boosting GABA release, and higher GABA inhibits the HPA axis. It also indirectly suppresses the HPA axis by boosting GABA release and stimulating hippocampal formation (Vitte et al., 1996; Cuthbert et al., 2000; Herman et al., 2004; Cullinan et al., 2008; Mody and Maguire, 2012), resulting in a spike in BDNF and GDNF levels. Bilateral CVS has been shown to improve different aspects of brain function in neurodegenerative pathologies (Devi and Mukkadan, 2017). However, only few research studies have been conducted to investigate the tangible impacts of bilateral CVS on CMS-induced rats. In the current work we have employed CVS to ameliorate behavior and neurotransmitters such as BDNF and GDNF levels in CMS-induced rats and it must be presumably owing to symmetrical stimulation of the cerebral hemispheres (Bense et al., 2003). Previous studies established the benefit of CVS in enhancing auditory and visual response speed under stress (Rajagopalan et al., 2017).

The behavioral analysis of the current research indicated that rats exposed to CMS, displayed a considerable decline in the locomotor count by actophotometer, muscle grip strength by hanging wire grip strength test, and immobilization time by forced swimming test. These behavioral aberrations in the CMS rats were recognized as a behavior of stress. The rats that were treated with CVS reversed the behavioral alterations which is obvious from the significant restoration of the locomotor count, muscle grip strength, and immobilization time, providing considerable protection for the neurons. These were ascribed as favorable effects of CVS in the inhibition of stress axis. These findings are in agreement with prior studies supporting the decrease in the depression, anxiety, and stress ratings followed by CVS (Kumar et al., 2016; Rajan et al., 2016).

The forced swimming test is performed in this study to assess immobilization. Immobility in a forced swimming test represents behavioral despair caused by the realization that escape is unattainable. The forced swimming test has also been used to assess active coping strategies, with immobility suggesting a passive coping response (Lam et al., 2018; Molendijk and de Kloet, 2019). CMS animals showed lower immobilization in the current study, indicating that the animals were less capable of coping with inescapable stressors, whereas CVS prevented CMS-induced changes in coping with inescapable stressors.

In the rotarod experiment, CMS animals showed a decrease in fall off time when compared to control animals, however the CMS animals co-administered with CVS prevented the CMS-induced alterations. The rotarod test is employed to evaluate the motor coordination of rodents and to detect cerebellar dysfunction (Shiotsuki et al., 2010). The decrease in locomotion in CMS group implies that the animal lost motor coordination due to stress which was prevented by CVS.

BDNF is generated from BDNF pro-isoform, which is then cleaved proteolytically (N-terminal domain is deleted) within the neuron or after it is released, forming its final protein form (Ahmed et al., 2015). This mature neurotrophin binds to protein-kinase neurotrophin receptors - tropomyosine-related kinase (Trk) receptors. The immunohistochemistry screening of hippocampal slices exhibiting BDNF and GDNF demonstrated substantial deterioration following CMS induction. Stress, diet, metabolism, and behavior modulates the expression of BDNF in the central and peripheral neural systems (Fuchikami et al., 2009). Stress results in morphological changes (McEwen, 2001), dendritic atrophy in hippocampal pyramidal neurons, particularly in the CA3, CA4 area, and an impairment of neurogenesis in the dentate gyrus (McEwen, 1999; Fuchs et al., 2001; Gill and Grace, 2013), as well as motor cortex thinning (Khan et al., 2018). Our present study also proves that stress causes pathological and morphological changes in the hippocampus, which is consistent with prior studies that show that several brain related issues, such as stress, seizure, ischemia, and hypoglycemia, alter BDNF expression in the central nervous system (Yan et al., 1997; Tapia-Arancibia et al., 2004). Changes in its expression may have a role in several disorders, including depression, Alzheimer’s disease, Parkinson’s disease, and epilepsy (Tapia-Arancibia et al., 2004). The treatment group (CMS+CVS) animals had higher levels of BDNF and GDNF, followed by the CVS group of rats. The surge in BDNF levels implies vestibular neurogenesis and modification of potassium-chloride cotransporter (KCC2) and GABA receptor expression in the vestibular nuclei. By boosting neurogenesis and modifying the expression of KCC2 and GABA receptors in the vestibular nuclei, BDNF signaling enhances vestibular compensation. The neurotrophic effects of GDNF against neuronal dysfunction are well documented. This GDNF neuroprotective action generated a powerful upregulation of BDNF, suggesting that together may crucial against neurons atrophy and degeneration (Revilla et al., 2014). There is a definite connection between the vestibular system and the brain. The vestibular system is intricately linked to the cerebral cortex, and vestibular system lesions trigger cortical and hippocampal atrophy (Brandt et al., 2005). Previous research has shown that bilateral loss of vestibular function is attributed to decrease hippocampal volume, cell number, proliferation, dendritic length, and morphology, all of which contribute to memory loss, anxiety, and autonomic dysfunction (Smith et al., 2010; Smith and Darlington, 2013; Balabhadrapatruni et al., 2016). Controlled CVS accelerates dendritic arborization in hippocampal pyramidal cells and boosts cell proliferation in the dentate gyrus and perhaps neurogenesis (Cuthbert et al., 2000; Khan et al., 2018). Vestibular stimulation impacts the physiology of the cortex due to its extensive interactions with brain regions. The current research presents data on stress-induced hippocampal morphological alterations, as well as the impact of CVS on stress-induced changes.

## Conclusion

The findings of the current study indicated that Chronic Mild Stress for 28 days induces behavioral and immunohistochemical modifications which is a significant indicator of neurodegeneration. The results further indicate that Caloric Vestibular Stimulation has ameliorating impact on Chronic Mild Stress with its therapeutic potential and can serve as a neuroprotectant in the treatment of stress-related disorders (Jinu and Archana, 2018), (Sailesh et al., 2014).

## Data Availability

The original contributions presented in the study are included in the article/Supplementary Materials, further inquiries can be directed to the corresponding authors.

## Footnotes

1
https://www.singlecare.com/blog/news/stress-statistics/
